# Identification of Novel Genes Involved in *Escherichia coli* Persistence to Tosufloxacin

**DOI:** 10.3389/fcimb.2020.581986

**Published:** 2020-09-30

**Authors:** Tuodi Li, Juan Wang, Qianqian Cao, Fei Li, Jiangyuan Han, Bingdong Zhu, Ying Zhang, Hongxia Niu

**Affiliations:** ^1^Institute of Pathogenic Biology, School of Basic Medical Sciences, Lanzhou University, Lanzhou, China; ^2^Department of Molecular Microbiology and Immunology, Bloomberg School of Public Health, Johns Hopkins University, Baltimore, MD, United States

**Keywords:** *Escherichia coli*, persistence, tosufloxacin, persister, molecular mechanism

## Abstract

Persisters are metabolically quiescent phenotypic variants of the wild type that are tolerant to cidal antibiotics, and the mechanisms of persister formation and survival are complex and not completely understood. To identify genes involved in persistence to tosufloxacin, which has higher activity against persisters than most other quinolones, we screened the *E. coli* KEIO mutant library using a different condition from most persister mutant screens (6 h) with a longer exposure of 18 h with tosufloxacin. We identified 18 mutants (*acrA, acrB, ddlB, dnaG, gltI, hlpA, lpcA, recG, recN, rfaH, ruvC, surA, tatC, tolQ, uvrD, xseA*, and *ydfI*) that failed to form tosufloxacin tolerant persisters. Among them, g*ltI, hlpA, ruvC, ddlB, ydfI*, and *tatC* are unique genes involved in *E. coli* persistence to tosufloxacin which have not been reported before. Furthermore, deletion mutants in genes coding periplasmic proteins (*surA, lpcA, hlpA*, and *gltI*) had more defect in persistence to tosufloxacin than the other identified mutants, with *surA* and *lpcA* mutants being the most prominent. The “deep” persister phenotype of *surA* and *lpcA* mutants was further confirmed both *in vitro* and *in vivo*. Compared with the wild type strain *E. coli* BW25113 *in vitro*, the persister phenotype of the *surA* and *lpcA* mutants was decreased more than 100–1,000-fold in persistence to various antibiotics, acidic, hyperosmotic and heat conditions. In addition, in both stationary phase bacteria and biofilm bacteria infection mouse models, the *surA and lpcA* mutants had lower survival and persistence than the parent uropathogenic strain UTI89, suggesting that the *in vitro* identified persister mechanisms (*surA* and *lpcA*) are operative and valid for *in vivo* persistence. Our findings provide new insight into the mechanisms of persister formation and maintenance under tosufloxacin and will likely provide novel therapeutic and vaccine targets for developing more effective treatment and prevention of persistent *E. coli* infections.

## Introduction

Persisters are a sub-population of genetically drug susceptible quiescent cells that are tolerant to cidal stresses (antibiotic, drug, heat, acidic pH and oxidative, etc.) and revive to metabolically active bacteria upon exiting stresses (Zhang, 2014). Persisters are implicated in many persistent bacterial infections, including tuberculosis, Lyme disease, urinary tract infections, and biofilm infections (Lewis, 2005; Stricker and Johnson, 2011; Zhang et al., 2012). As the quiescent persister bacteria have the capacity to survive antibiotics and revert to replicating forms, persisters are responsible for relapse after treatment.

Persisters have been regarded as phenotypic variants of the wild type that are not all the same. As expressed in a Yin-Yang model (Zhang, 2014), persisters have varying hierarchy and enormous heterogeneity, such as “shallow” persisters or “deep” persisters. The level and proportion of persister formation are affected by many factors, including bacterial age, environments that the bacteria reside in, and antibiotic exposure (Joers et al., 2010; Zhang, 2014). Due to its complexity, the mechanism of persister formation and survival are not completely understood. Mutagenesis screens have been traditionally used to isolate genes involved in persister formation. For example, screening the *Escherichia coli* KEIO mutant library identified several mutants (*relA, phoU, sucB*, and *ubiF*, etc.) with reduced antibiotic tolerance in different studies (Korch et al., 2003; Li and Zhang, 2007; Ma et al., 2010). It is observed that different persister genes can be identified at different exposure conditions of screening, including antibiotic type, concentration and exposure length.

We previously found that tosufloxacin (50 μM) compared with other quinolones had higher activity against both *Escherichia coli* (*E. coli*) and *Staphylococcus aureus* (*S. aureus*) persisters *in vitro* (Niu et al., 2015a,b), but it could not kill the biofilm persisters *in vivo* (unpublished observation). However, it is not clear what determines its unique activity against bacterial persisters. To identify genes involved in persistence to tosufloxacin, we screened the *E. coli* KEIO mutant library with tosufloxacin and identified several interesting candidate genes that encode periplasmic proteins SurA, LpcA, HlpA, and GltI whose mutations caused reduced persistence to tosufloxacin.

## Materials and Methods

### Bacterial Strains, Antibiotics, and Cell Strain

Uropathogenic *E. coli* strain UTI89, the KEIO collection of *Escherichia coli* K-12 single-gene knockout mutants and its parent strain BW25113 were used in this study. All the strains were cultured in Luria-Bertani (LB) medium. Tosufloxacin, levofloxacin, ofloxacin and gentamicin were purchased from Sigma-Aldrich Chemical Co. (St. Louis, MO, USA) and were used at different concentrations (see below).

Human bladder epithelial cell strain (ATCC HTB-9) was purchased from Bio-feng company (Shanghai, China) and was cultured in RPMI 1640 with 10% newborn calf serum.

### Animals

C57BL/6 or Bab/c female mice (6–8 weeks old) were obtained from Lanzhou Veterinary Research Institute and maintained in clean animal facility at Lanzhou University. Animals received free access to water and standard mouse chow throughout the study. The use of animals was in compliance with the guidelines set by the Institutional Animal Care and Use Committee of Lanzhou University.

### Screening for Mutants With Defective Persistence to Tosufloxacin

The 3,985 single-gene knockout mutants in the *E. coli* KEIO mutant library were arrayed in 46 96-well plates. Then, the mutants were transferred to 200 μl fresh LB broth containing 30 μg/mL kanamycin using a 96-pin replicator and cultured overnight at 37°C without shaking. The overnight culture (~3 × 10^9^ CFU/mL cells) was exposed to tosufloxacin (10 μM, 333 × MIC) for 18 h. Finally, bacteria were plated onto LB agar plates using 96-pin replicator and mutants with defective persistence, as indicated by the lack of growth, were identified for further analysis. The active hits were confirmed by rescreens using the same procedure for three times.

### Comparison of the Persistence of the Identified Mutants by Time-Kill Curve Studies

The identified mutants with defective persistence were cultured to stationary phase and dispensed into 1.5 mL Eppendorf tubes in 1 mL followed by addition of tosufloxacin (10 μM, 333 × MIC). Each day after exposure, 100 μl of the culture samples was taken out, washed with phosphate buffered saline (PBS), serially 10-fold diluted and plated for colony formation unit (CFU) count on LB agar plates.

### Complementation of *E. coli* Mutants

To complement the gene knockout mutants of interest (Δ*lpcA* and Δ*surA*), plasmids encoding *lpcA* and *surA* were generated by inserting the gene fragments into the multiple cloning sites of pTrc99a separately as follows. Initially, the DNA sequences of *lpcA* and *surA* were generated by PCR amplification from the DNA of wild type strain with primers *lpcA* A-F and *lpcA* A-R, *surA* A-F, and *surA* A-R (Table 1). Then the fragments were cloned into the unique sites of pTr99a plasmid to obtain the recombinant plasmids pTrc99a-*lpcA* and pTrc99a-*surA*. Finally, the recombinant plasmids or empty vector pTrc99a were transformed into the corresponding mutants to construct complementation strains.

**Table 1:** Primers used in this study.

**Primer name**	**Sequence (5'-3')**
*lpcA* A-F	CCG*GAATTC*ATGCGTAACGAACTGAACGAAGC (*Ecor* I)
*lpcA* A-R	CGC*GGATCC*TTATTTTCAATCAACTGGATCAGGA (*BamH* I)
*surA* A-F	CGT*GAATTC*ATGAAGAACTGGAAAACGCTGCTTC (*Ecor* I)
*surA* A-R	TCG*AAGCTT*AGTTGCTCAGGATTTTAACGTAG (*Hind* III)
*surA* B-F	AATCCCGGCGGGCTCGCCGGGAGTGATCACAACACGTTGGG TTTTAAGTGTAGGCTGGAGCTGCTTC
*surA* B-R	TGTTGATTTACCACGTAATCCGCAGTGCGGTTAATTGAAATGGA AAAAGTATGGGAATTAGCCATGGTCC
*lpcA* B-F	ATCCGGTACACTGCATTTTGTCTATTACATTTATGCTGAAGGATA TCCTCCCAGTGTAGGCTGGAGCTGCTTC
*lpcA* B-R	CGCACAAATGCCGGATGCGGCGTAAACGTCTTATCCGGCCTAC GCCAGACATGGGAATTAGCCATGGTCC
*surA* C-F	ATGAAGAACTGGAAAACGCT
*surA* C-R	TTAGTTGCTCAGGATTTTAAC
*lpcA* C-F	CGTAACGAACTGAACGAAGC
*lpcA* C-R	TTTTCAATCAACTGGATCAG

*The sequences of restriction endonucleases are italicized. The FRTs (flippase recognition targets) flanked by chloramphenicol resistance genes are underlined*.

### Evaluation the Persistence of Bacteria to Antibiotics and Various Stresses

*E. coli* wild type strain BW25113, mutants (Δ*lpcA* and Δ*surA*) and complemented strains (Δ*lpcA-pTrc99a-lpcA* and Δ*surA-pTrc99a-surA*) were all cultured overnight to stationary phase, and then treated with tosufloxacin (10 μM), levofloxacin (10 μM), ofloxacin (10 μM), and gentamicin (20 μM). Three days post exposure, the surviving bacteria were determined by CFU counting.

For acidic, hypertonic and heat stresses assays, stationary phase cultures were washed and diluted 100-fold, and then exposed to acidic condition (pH3.0 LB broth) for 2 days, hyperosmotic condition (3M NaCl LB broth) for 2 days, or heat condition (52°C water bath) for 3 h. Surviving bacteria were also determined by CFU counting.

### Generation of Gene Knockout Strains in *E. coli* UTI89

Uropathogenic *E. coli* UTI89 mutant strains Δ*surA* and Δ*lpcA* were generated using λ red homologous recombination system (Datsenko and Wanner, 2000). Firstly, linear fragment of FRT (flippase recognition target)-flanked chloramphenicol resistance gene were generated by PCR from pKD3 using primers *surA* B-F and *surA* B-R, *lpcA* B-F and *lpcA* B-R (Table 1). Then, pKD46 carrying the λ red recombinase gene was transformed into *E. coli* UTI89, and the transformants were grown in 5-mL LB broth with ampicillin and L-arabinose at 30°C to an OD600 of 0.4 and then made electrocompetent cells by concentrating and washing three times with ice-cold 10% glycerol. Next, the amplified linear fragments were transformed into the electrocompetent cells using Bio-Rad Gene Pulser Xcell™. The shocked cells were added to 1-mL LB, incubated 2 h at 37°C, and then the culture was spread onto LB agar with chloramphenicol to select Cm-resistant tansformants. Finally, PCR verify the deletion with primers *surA* C-F and *surA* C-R, *lpcA* C-F, and *lpcA* C-R (Table 1).

### Biofilm Formation Assay in Microtiter Dish

The biofilm formation of uropathogenic *E. coli* UTI89 and its mutant strains Δ*surA* and Δ*lpcA* was investigated in microtiter plates as described (O'Toole, 2011). Briefly, uropathogenic *E. coli* UTI89 and the mutant strains were cultured overnight in 10 mL LB broth at 37°C, and then the bacterial culture was diluted 1:100 into fresh LB broth for biofilm assay. The dilutions were added in a 96 well dish (100 μL/well) with 8 replicate wells for each strain, and the microtiter plate was incubated for 24 h at 37°C. After incubation, each well was washed twice with PBS, added crystal violet solution (0.1%) and incubated for 10 min. Next, the biofilm stain was dissolved with 30% acetic acid and quantified absorbance in a plate reader at 550 nm.

### UTI Mouse Model

The persistence of uropathogenic *E. coli* UTI89 mutant strains Δ*surA* and Δ*lpcA in vivo* were analyzed in UTI mouse models (Hung et al., 2009). Firstly, C57BL/6 or Balb/c mice were anesthetized with 4% chloral hydrate (10 μL/g) by intraperitoneal injection. Then, the mice were inoculated with 50 μL stationary phase bacteria or biofilm bacteria (10^7^ CFU per mice) via transurethral route. For stationary phase inoculum, bacteria were cultured over 24 h, and then were washed and resuspended in PBS prior to injection into C57BL/6 mice. For biofilm inoculum (O'Toole, 2011; Yee et al., 2019b), biofilms were first grown in LB medium in 96-well plates. After 24 h of incubation, the planktonic cells were removed, and the wells were washed with PBS. The biofilms in the bottom or the side wall of the wells were mechanically dislodged by scraping the bottom of the microtiter well using a pipette and resuspended in PBS. Quantification of all inoculum was performed by serial dilution and plating. On the 1st, 3rd, 5th, or 6th day post infection, mice were sacrificed and the whole bladder or kidney were harvested and homogenized aseptically. Finally, the individual homogenate was serial diluted with sterile PBS and plated on LB agar in triplicates for CFU count.

### Adhesion and Invasion Assays in Bladder Epithelial Cells

Adhesion and invasion assays were performed as described (Elsinghorst, 1994; Martinez et al., 2000). Firstly, human epithelial cells (ATCC HTB-9) were seed in 90 mm plates in 500 μL of RPMI 1640 with 10% newborn calf serum, and single-cell suspension of the epithelial cells was prepared by trypsinization. The suspension was diluted in fresh, pre-warmed 1640 medium, and the diluted cell suspension was added to a 24-well plate at 2 × 10^6^ cell in 1 mL per well. *E. coli* UTI89 and its mutant strains Δ*surA* and Δ*lpcA* were cultured overnight and diluted l: 1000 into fresh LB broth and grown at 37° to mid-log phase (OD600 = ~0.5). The mid-log phase bacteria were then centrifuged, resuspended in PBS and adjusted to appropriate concentration according to the results of CFU count. Next, epithelial cells were infected with bacteria in a multiplicity of infection (m.o.i.) of 5~10 per cell in three sets of triplicate wells. After 2 h co-incubation at 37°C, one set of the infected cells was lysed with 0.1% Triton X-100, and the lysates were plated on LB-agar plates to calculate the total number of infected bacteria (represented as A).

For adherence assays, a second set of cells were firstly washed five times with PBS, lysed with 0.1% Triton X-100, and then the lysates were plated on LB-agar plates to calculate the number of bacteria recovered after PBS washes (represented as B). Adherence frequency was calculated as B/A. For invasion assays, after the initial 2 h co-incubation, a third set of cells were firstly washed twice with PBS and then continue to incubate for 2 h with gentamicin (100 μg/mL) in RPM-1640 to kill extracellular bacteria. Cells were then washed additional three times with PBS, lysed with 0.1% Triton X-100, and plated on LB-agar plates to calculate the number of bacteria surviving incubation with gentamicin (represented as C). Invasion frequency was calculated as C/B.

### Statistical Analysis

All the experiments in this study were done in triplicates and repeated for three times, and the results were expressed as means ± SD. The significance of experimental differences between the mutants and the wild type strains was evaluated by unpaired Student's *t*-test using SPSS13.0 software. Values of *p* < 0.05 were considered as statistically significant.

## Results

### Identification of Mutants With Defective Persistence to Tosufloxacin

To better understand the genes involved in persistence to tosufloxacin, we performed a genetic screen using the *E. coli* KEIO collection which contained 3,985 single-gene knockout mutants. Screening procedure and analysis are shown in methods. The overnight culture was exposed to tosufloxacin (10 μM), which has the highest bactericidal activity among different quinolones for *E. coli* (Hongxia et al., 2018), for 18 h. Using a 96-pin replicator, bacteria were stamped onto LB agar plates and mutants with defective persistence, as indicated by the lack of growth, were identified for further examination.

Of the 3,985 mutants in the *E. coli* KEIO collection, we identified 18 mutants (*acrA, acrB, ddlB, dnaG, gltI, hlpA, lpcA, recG, recN, rfaH, ruvC, surA, tatC, tolQ, uvrD, xseA*, and *ydfI*), that failed to grow on LB plates after exposure with tosufloxacin (10 μM) (Table 2). The target mutants were selected and rescreened twice to confirm a stable phenotype with defective persistence to tosufloxacin (10 μM). Of the mutant genes in the 18 identified mutants, *dnaG, recG, recN, rfaH, ruvC, xs*e*A*, and *xseB* play a role in genetic processes such as DNA replication and repair; *ddlB, ydfI*, and *tatC* are enzymes (e.g., protein translocase, ligase or oxidoreductase); *gltI, lpcA, hlpA*, and *surA* are periplasmic proteins; *acrA* and *acrB* are acriflavine efflux proteins; *tolQ* is an outer membrane protein involved in the integrity of the bacterial envelope and the import of both filamentous phage and group A colicins.

**Table 2:** Function of the identified 18 genes involved in tosufloxacin persistence.

**Mutants**	**Gene function**
*acrA*	Acriflavine resistance protein A
*acrB*	Acriflavine resistance protein B
*ddlB*	D-alanine-D-alanine ligase B
*dnaG*	DNA primase
*gltI*	Glutamate/aspartate periplasmic-binding protein
*hlpA*	Periplasmic molecular chaperone
*lpcA*	Phosphoheptose isomerase
*recG*	ATP-dependent DNA helicase
*recN*	DNA repair protein
*rfaH*	Transcriptional activator
*ruvC*	Crossover junction endodeoxyribonuclease
*surA*	Periplasmic molecular chaperone
*tatC*	Sec-independent protein translocase protein
*tolQ*	an out-membrane protein
*uvrD*	DNA helicase II
*xseA*	Exodeoxyribonuclease 7 large subunit
*xseB*	Exodeoxyribonuclease 7 small subunit
*ydfI*	Uncharacterized oxidoreductase

#### The surA and lpcA Mutants Had the Most Defect in Persistence to Tosufloxacin

**Figure 1:**
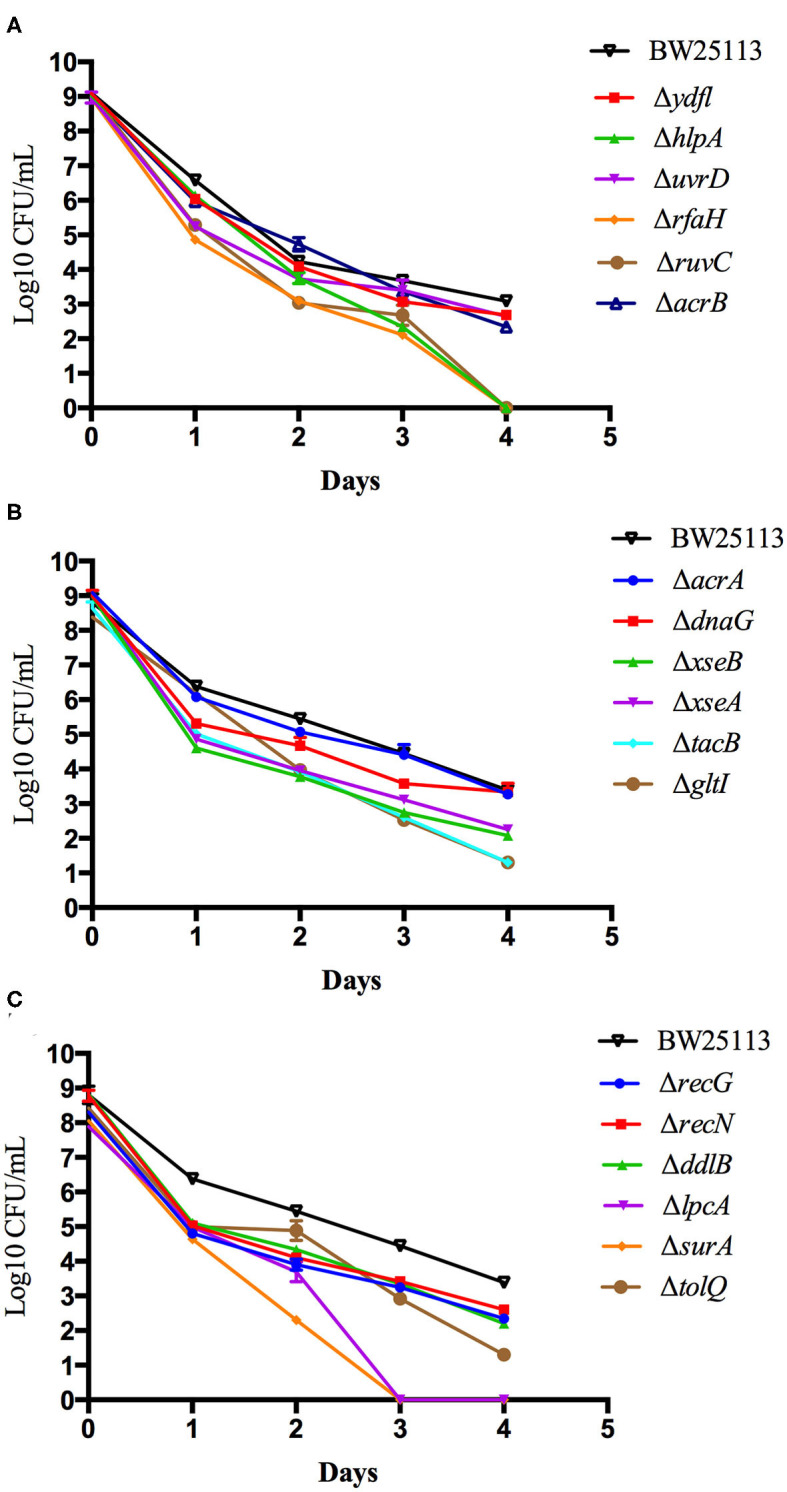
Compare the persistence ability of the identified mutants by CFU count. **(A)** Tosufloxacin killing curve of ydfl, hlpA, uvrD, rfaH, ruvC, acrB mutants. **(B)** Tosufloxacin killing curve of acrA, dnaG, xseB, xseA, tacB, gltI mutants. **(C)** Tosufloxacin killing curve of recG, recN, ddlB, lpcA, surA, tolQ mutants. Grow the identified mutants and their parent strain *E. coli* BW25113 in LB broth medium in tube. The cultures were grown to stationary phase (final concentration, >1 × 10^9^ CFU/mL) and treated with tosufloxacin (10 μM) for the indicated days. Samples were diluted and spot plated on LB agar per day. The experiments were done in triplicates and repeated for three times. The results are expressed as means ± SD.

To further validate and compare the persistence ability of the identified mutants above, we performed time-dependent killing studies as described in methods. Compared with the wild-type BW25113 strain, all the mutants exhibited increased susceptibility and defective persistence to tosufloxacin (10 μM) at various time points. However, after only 3 days of tosufloxacin (10 μM) exposure, there were no surviving cells in *surA* and *lpcA m*utants, whereas more than 10^2^ CFU/mL bacteria still remained in the rest of the mutants and 10^5^-10^6^ CFU/mL bacteria in the wild-type BW25113 strain (Figures 1A–C). Next, *gltI, hlpA, tatC, rfaH, ruvC* mutants, died completely 4 days post tosufloxacin exposure (10 μM) (Figures 1B,C). The results showed that the *surA* and *lpcA* mutants were the most susceptible and had most defect in persistence to tosufloxacin.

### Survival of *surA* and *lpcA* Mutants and Their Complemented Strains to a Variety of Stresses Including Antibiotics, Acidic, Hypertonic, and Heat Conditions

To further validate the role of *surA* and *lpcA* in persistence, the *surA* and *lpcA* mutants were complemented with their wild type gene using vector pTrc99a. Then we exposed all the mutants and their complemented strains and control strain to antibiotics including tosufloxacin, gentamicin, levofloxacin, ofloxacin and more stress conditions such as acidic, hypertonic and heat conditions.

**Figure 2:**
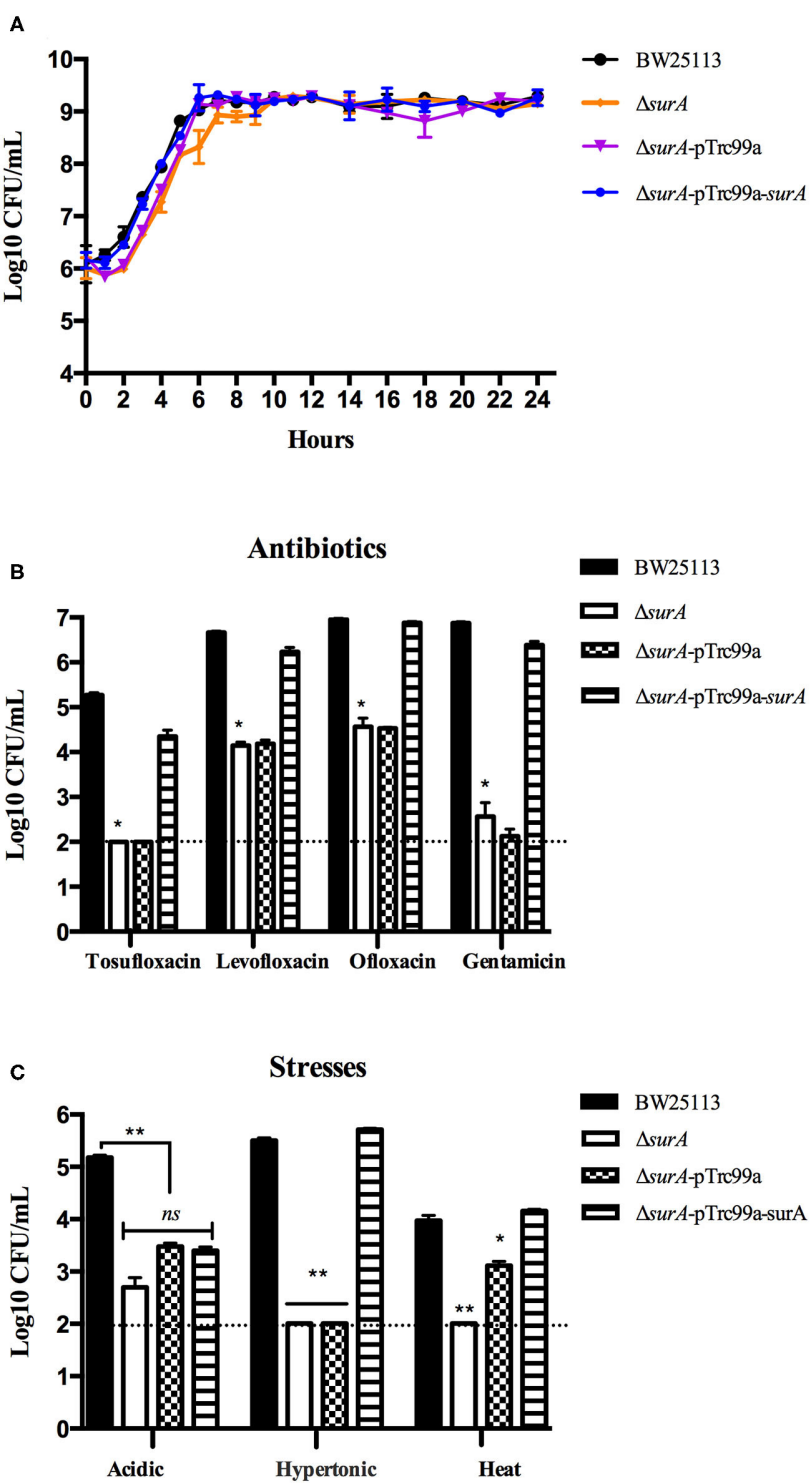
Survival of *E. coli* mutantΔ*surA* and its complemented strain exposed to different antibiotics and stress conditions. **(A)** Growth curve of the wild type strain *E. coli* BW25113, mutant strain Δ*surA*, and complemented strain Δ*surA* transformed with plasmid pTrc99a-*surA*. **(B)** Persistence ability of the stationary phase culture to antibiotics. All the strains were cultured overnight and then treated with tosufloxacin (10 μM), levofloxacin (10 μM), ofloxacin (10 μM), and gentamicin (20 μM) for 3 days. Survival of bacteria was determined by CFU counting. **(C)** Persistence ability of the stationary phase cultures to acidic, hypertonic and heat stresses. The stationary phase culture was diluted 100-fold and then exposed to acidic condition (pH3.0 LB broth) for 2 days, hyperosmotic condition (3M NaCl LB broth) for 2 days, or heat condition (52°C water bath) for 3 h. Survival of bacteria was determined by CFU counting. The experiments were done in triplicates and repeated for three times. The results are expressed as means ± SD. *p < 0.05, relative to BW25113 and Δ*surA*-pTrc99a-*surA* groups. **p < 0.01, relative to BW25113 and Δ*surA*-pTrc99a-*surA* groups. *ns* means no significance between the groups of Δ*surA* and Δ*surA*-pTrc99a-*surA*.

**Figure 3:**
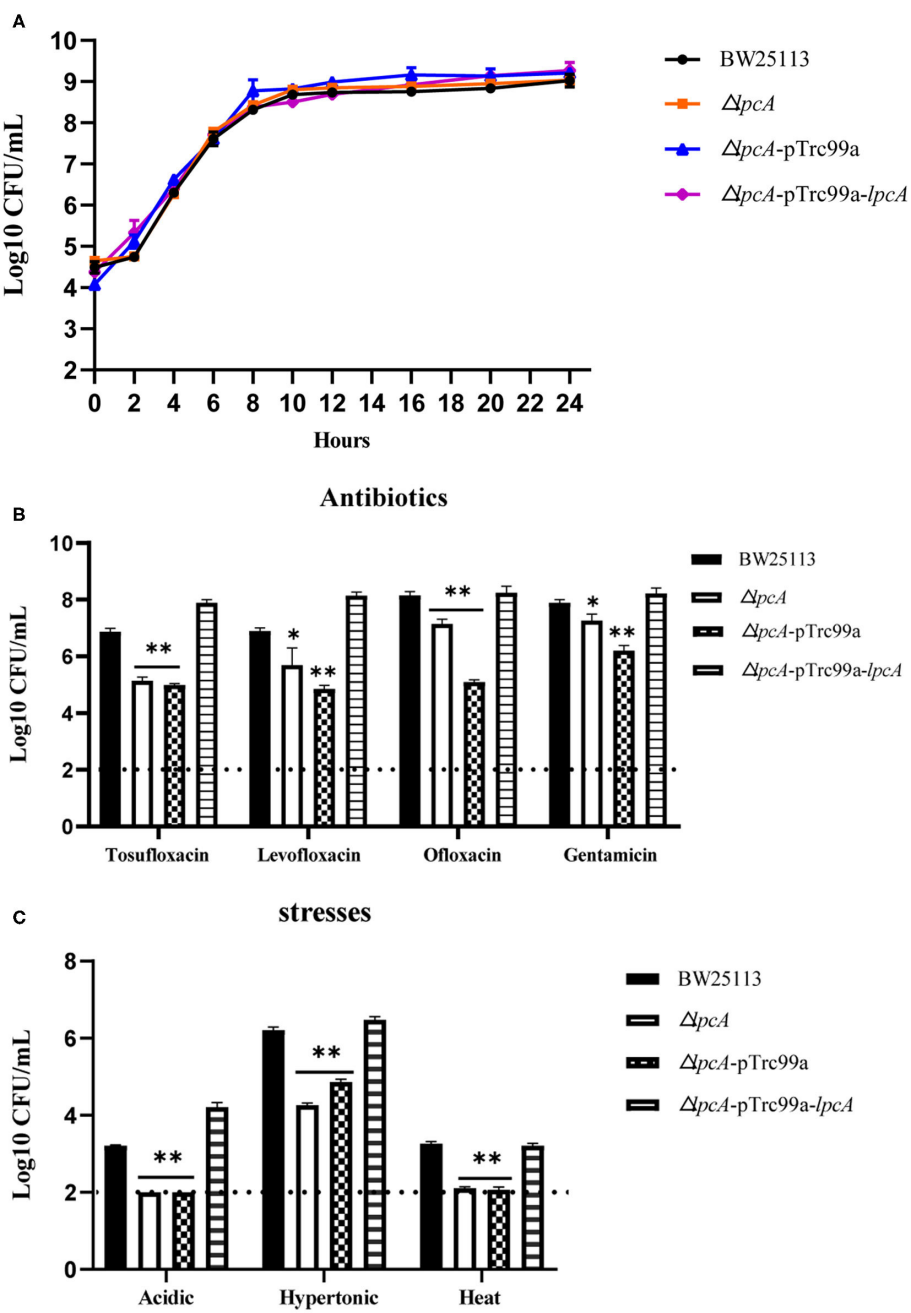
Survival of *E. coli* mutant Δ*lpcA* and its complemented strain exposed to different antibiotics and stress conditions. **(A)** Growth curve of the wild type strain *E. coli* BW25113, mutant strain Δ*lpcA*, and the complemented strain Δ*lpcA* transformed with plasmid pTrc99a-*lpcA*. **(B)** Persistence ability of the stationary phase culture to antibiotics. All the strains were cultured overnight and then treated with tosufloxacin (10 μM), levofloxacin (10 μM), ofloxacin (10 μM), and gentamicin (20 μM) for 3 days. Survival of bacteria was determined by CFU counting. **(C)** Persistent ability of the stationary phase cultures to acidic, hypertonic and heat stresses. The stationary phase culture was diluted 100-fold and then exposed to acidic condition (pH3.0 LB broth) for 2 days, hyperosmotic condition (3M NaCl LB broth) for 2 days, or heat condition (52 °C water bath) for 3 h. Survival of bacteria was determined by CFU counting. The experiments were done in triplicates and repeated for three times. The results are expressed as means ± SD. *p < 0.05, relative to BW25113 and Δ*surA*-pTrc99a-*surA* groups. **p < 0.01, relative to BW25113 and Δ*surA*-pTrc99a-*surA* groups.

Before the persistence assay, we first conducted a growth curve study by CFU counts to exclude the possibility that *surA* and *lpcA* mutants have any growth defects. The results showed that, under non-stress conditions, *surA* and *lpcA* mutants had the same growth ability in log phase and stationary phase compared with BW25113 strain (Figures 2, 3A). We then performed an MIC experiment for tosufloxacin, ciprofloxacin, ofloxacin, gentamicin and fosfomycin with these two mutants. Compared with control strain BW25113 strain, s*urA* and *lpcA* mutants showed at least 4-folds decrease in MICs to tosufloxacin, while *lpcA* mutant had the same MICs to ciprofloxacin, ofloxacin, and s*urA* mutant showed 2-fold decrease of MICs to ciprofloxacin, ofloxacin. And all the mutants had the same MICs to gentamicin and fosfomycin as BW25113 strain (Table 3).

**Table 3:** The MICs of antibiotics (tosufloxacin, ciprofloxacin, oflofloxacin, gentamycin and fosfomycin) against *E. coli* BW25113 and its mutants Δ*lpcA* and Δ*surA*.

**Strains**	**Tosufloxacin (μM)**	**Ciprofloxacin (μM)**	**Ofloxacin (μM)**	**Gentamicin (μM)**	**Fosfomycin (μM)**
BW 25113	0.03 ± 0.008	0.03 ± 0.008	0.06	8	14
Δ*lpcA*	<0.0075	0.03 ± 0.008	0.06	8	14
Δ*surA*	<0.0075	0.0125 ± 0.004	0.03 ± 0.008	8	14

*The test antibiotics were subjected to 2-fold serial dilutions in the 96-well plate in triplicates for each strain tested. The results are shown as means of three replicates ± SD*.

Next, we performed the persistence assays, where stationary-phase bacteria were exposed to different stresses for different times separately, including treatment with tosufloxacin (10 μM), levofloxacin (10 μM), ofloxacin (10 μM) and gentamicin (20 μM) for 3 days, exposure to acidic condition (pH3.0 LB broth) for 2 days, hyperosmotic condition (3M NaCl LB broth) for 2 days, or heat condition (52°C water bath) for 3 h. *surA* mutant showed increased susceptibility and defective persistence to all the antibiotics (tosufloxacin, ciprofloxacin, ofloxacin and gentamicin), while its complemented strain Δ*surA-pTrc99a-surA* restored the persistence level to that of the wild type strain (Figure 2B). When exposed to hyperosmotic and heat conditions, upon each time point, the surviving cells in the *surA* mutant was below the detection limit (10 CFU/mL), whereas the wild type strain BW25113 had more than 10^5^ CFU/mL bacteria left in the hyperosmotic and 10^4^ CFU/mL bacteria remained in heat treatment (Figure 2C). Under acidic condition, the remaining bacteria of the *surA* mutants was down to 10^2^ CFU/mL, whereas wild type strain BW25113 had roughly 10^5^ CFU/mL bacteria upon 2 days post exposure (Figure 2C). In addition, the persister levels of the complemented strain Δ*surA-pTrc99a-surA* were restored to that of the wild type strain in hyperosmotic and heat conditions (Figures 2B,C).

Consistent with the *surA* mutant, *lpcA* mutant also displayed increased susceptibility and defective persistence to all the stresses (antibiotics, acidic, hypertonic and heat conditions) in different persistence assays, especially to acidic and heat conditions (Figures 3B,C). And, the persistence ability of its complemented strain Δ*lpcA -pTrc99a-lpcA* was restored to that of the wild type strain BW25113 (Figures 3B,C).

The results indicate that both *surA* and *lpcA* mutants had significantly less persistence ability to different antibiotics and stress conditions than the wild type strain BW25113 (*p* < 0.05) (Figures 2, 3).

### Defective Biofilm Formation of Uropathogenic *E. coli* ΔsurA and ΔlpcA Mutants

**Figure 4:**
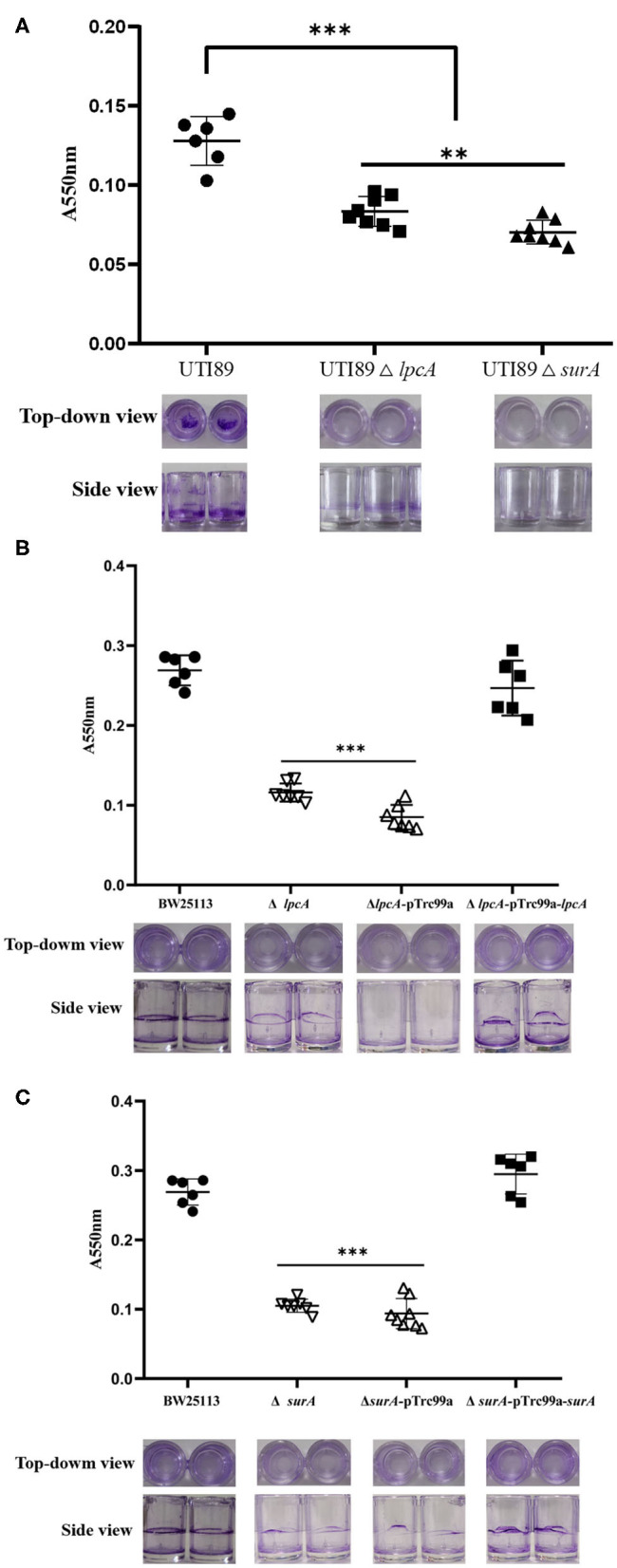
The biofilm formation level of *E. coli* Δ*surA* and Δ*lpcA* mutants in microtiter dish. **(A)** Uropathogenic *E. coli* UTI89 Δ*surA* and Δ*lpcA* mutants. **(B)** *E. coli* BW25113 Δ*surA* and its complemented strain. **(C)** *E. coli* BW25113 Δ*lpcA* and its complemented strain. *E. coli* UTI89, *E. coli* BW25113 and their mutant strains Δ*surA* and Δ*lpcA* were grown in a 96-well dish (100 μL/well) with 8 replicate wells for each strain, and the microtiter plate was incubated for 24 h at 37°C. Biofilm level was measured spectrophotometrically at 550 nm. Representative images of stained biofilms were shown at the bottom of the figure. The experiments were done with 8 replicates and repeated for three times. The results are expressed as means ± SD. **p < 0.01, compared with each other between UTI89 Δ*surA* and Δ*lpcA* groups. ***p < 0.001, relative to UTI89 group.

Persister cells can be found to be heavily enriched inside biofilms due to the high cell density, and nutrient and oxygen limiting environment inside the biofilm matrix (Kavanaugh and Horswill, 2016). To evaluate whether mutations in *surA* and *lpcA* influence the biofilm formation of *E. coli*, we did a biofilm formation assay in a microtiter plate. We observed that uropathogenic *E. coli* UTI89 was capable of adhering to the walls and bottoms of the microtiter plate wells to form biofilm. However, the mutant strains UTI89 Δ*surA* and Δ*lpcA* formed significant weaker biofilm than the wild type strain UTI89 (*p* < 0.001), with at least 50% reduction (Figure 4). Besides, mutant strain UTI89 Δ*surA* showed much less biofilm formation than Δ*lpcA* (*p* < 0.05).

### Persistence of Uropathogenic *E. coli* UTI89 ΔsurA and ΔlpcA Mutants in Mice

Our *in vitro* persister assays revealed that both *surA* and *lpcA* mutants had defect in persistence to antibiotics and stresses. To further evaluate the ability of the *surA* and *lpcA* mutants to survive and persist *in vivo*, we could not use the non-pathogenic strain *E. coli* BW25113 and instead we constructed mutant strains of Δ*surA* and Δ*lpcA* in uropathogenic *E. coli* UTI89 and evaluated their persistence ability in C57BL/6 and Bab/c mice. The C57BL/6 mice were infected with stationary phase persisters of *E. coli* UTI89 and its mutants Δ*surA* and Δ*lpcA*, and on the 1st, 3^rd^, and 5th day post infection, the bacterial loads in bladder and kidney of the infected mice were analyzed. The Bab/c mice were infected with biofilm persisters of *E. coli* UTI89 and its mutants Δ*surA* and Δ*lpcA*, and the bacterial loads in bladder, kidney and urine were analyzed 6 days after infection.

**Figure 5:**
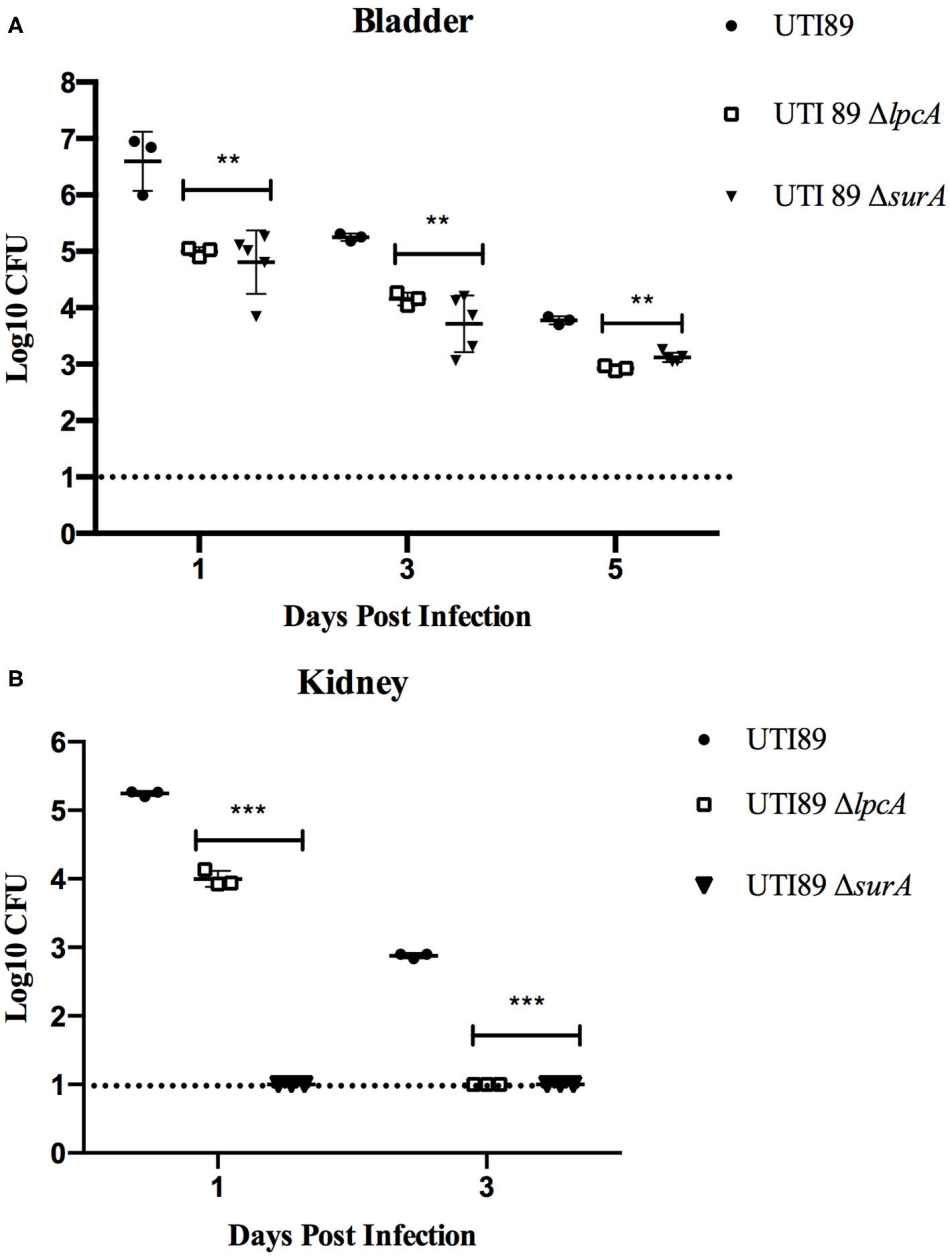
The persistence ability of stationary phase bacteria of uropathogenic *E. coli* UTI89 mutant strains Δ*surA* and Δ*lpcA* in C57BL/6 mice. **(A)** Bacterial in bladder. **(B)** Bacterial in kidney. The mice were infected with *E. coli* UTI89, UTI89 Δ*surA*, and UTI89 Δ*lpcA* (10^7^CFU per mice) via transurethral route, respectively. On the 1st, 3rd, and 5th day post infection, mice were sacrificed and their whole bladder and kidney were homogenized. Then, the individual homogenization was serial diluted with sterile PBS and plated on LB agar in triplicates for CFU count (limit of detection is 10 bacteria/sample). The results (CFU/sample) are expressed as means of at least 3 mice ± SD. **p < 0.01, relative to UTI89 group. ***p < 0.001, relative to UTI89 group.

In C57BL/6 mice infected with stationary phase persisters, the bacterial loads in bladder and kidney were decreased gradually as time went on post infection in general for different strains, though the mutant strains Δ*surA* and Δ*lpcA* had significant lower bacterial loads than their parental strain UTI89 (*p* < 0.01) (Figure 5). In bladder (Figure 5A), both the *surA* and *lpcA* mutants had ~10^3^ CFU on the 5th day post infection, whereas the parental strain had 10^4^ CFU. In kidneys (Figure 5B), on the 1st day post infection, no bacteria were detected in *surA* mutant infected mice, whereas 10^4^ CFU and 10^5^CFU bacteria were detected in *lpcA* mutant and UTI89 wild type strain infected mice separately; on the 3rd day post infection, the bacterial load in *lpcA* mutant decreased to the level below the detection limit, whereas UTI89 wild type strain still had 10^3^ CFU bacteria remaining. The results suggest that *surA and lpcA* mutants have lower persistence than the wild type strain UTI89. Besides, stationary phase *surA* mutant was incapable of infecting kidney (Figure 5B).

**Figure 6:**
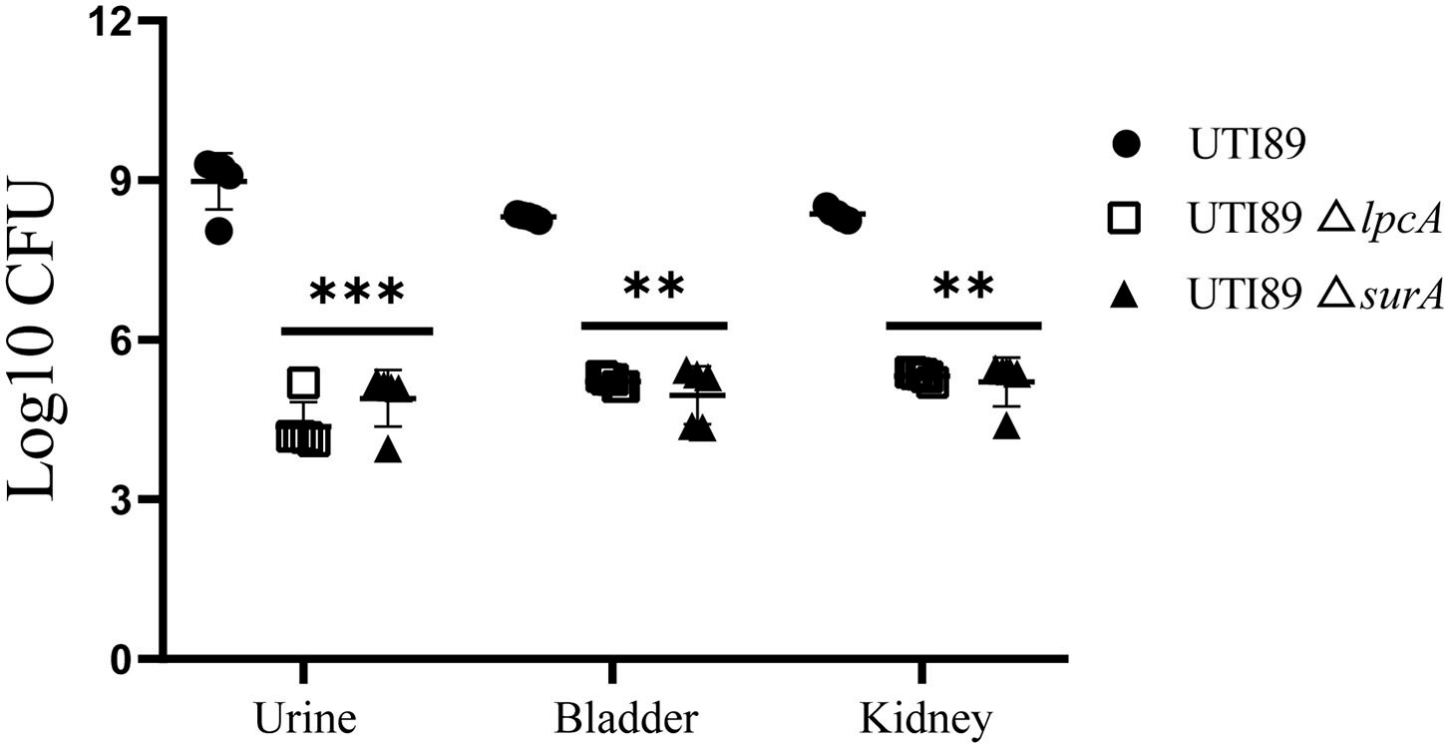
Defective persistence of the Δ*surA* and Δ*lpcA* mutant strains compared with uropathogenic *E. coli* UTI89 parent strain in Bab/c mice. The mice were infected with biofilm persisters of *E. coli* UTI89 and its mutants Δ*surA* and Δ*lpcA* (10^7^ CFU per mice) via transurethral route, respectively. On 6th day post infection, mice were sacrificed and their whole bladder or kidneys were homogenized, and the homogenization was serial diluted and plated on LB agar for CFU count. The urine was serial diluted and plated on LB agar for CFU count directly, and the result is shown as CFU/mL. The results (CFU/sample or CFU/mL for urine) are expressed as means of 5 mice ± SD. **p < 0.01, relative to UTI89 group. ***p < 0.001, relative to UTI89 group.

On 3rd or 6th days post infection, Bab/c mice infected with biofilm persisters harbored elevated bacterial loads compared to the mice infected with stationary phase persisters or log-phase bacteria (data not published), indicating that biofilm persisters are the most persistent form. In this biofilm persister infection mouse model, at 6 days after infection, the mice infected with mutant strains UTI89 Δ*surA* and Δ*lpcA* harbored 10^3^~10^4^ CFU in bladder, kidney or urine, whereas the mice infected with wild type strain UTI89 had at least 1,000-10,000-folds higher bacteria (*p* < 0.01) (Figure 6). These *in-vivo* findings suggest that the mutation in *surA* or *lpcA* caused defect in persistence not only *in vitro* but also *in vivo* in the mouse persister inocula model.

### The Adhesion and Invasion Abilities of Uropathogenic *E. coli* UTI89 ΔsurA and ΔlpcA Mutants in Bladder Epithelial Cells

**Figure 7:**
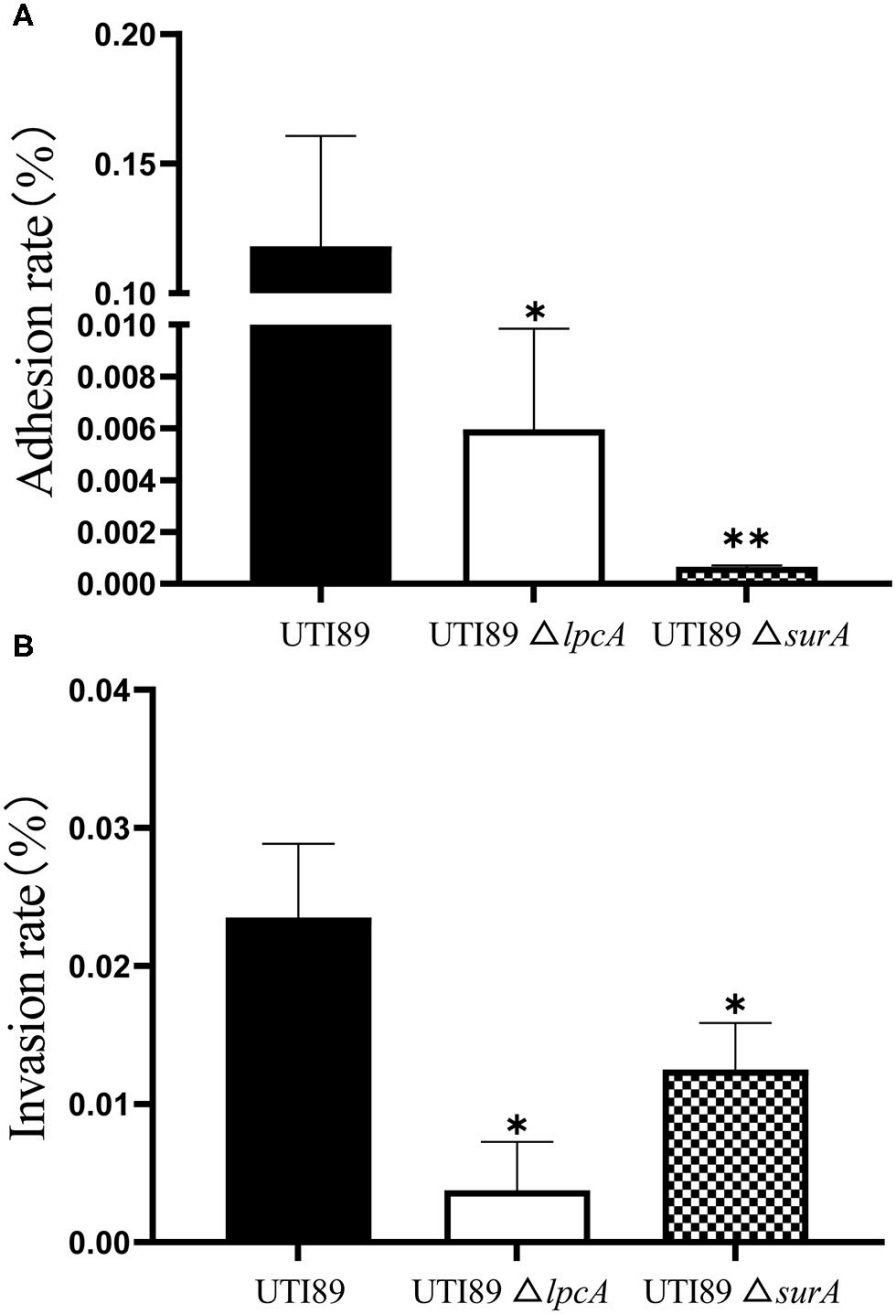
Defective adhesion and invasion of the Δ*surA* and Δ*lpcA* mutants compared with uropathogenic *E. coli* UTI89 in bladder epithelial cells. **(A)** The adhesion ability of mutant strains. **(B)** The invasive capability of mutant strains. The human epithelial cells (ATCC HTB-9) (2 × 10^6^ cell/well) were infected with log-phase *E. coli* UTI89 and its mutants Δ*surA* and Δ*lpcA* in a multiplicity of infection (m.o.i.) of 5~10 per cell in 24-well plate. At 2 h post infection, the adhesion and invasion frequencies were analyzed as described in Methods. The experiments were done in triplicates and repeated for three times. The results are expressed as means ± SD. *p < 0.05, relative to UTI89 group. **p < 0.01, relative to UTI89 group.

To determine whether *surA* and *lpcA* participate in mediating bacterial adhesion and invasion of host cells, we further did the adhesion and invasion assays in human bladder epithelial cells. We infected the epithelial cells with *E. coli* UTI89 and its mutants Δ*surA* and Δ*lpcA* for 2 h separately, and found that both the adhesion and invasion frequencies of the wild-type strain UTI89 were obviously higher than the mutant strains Δ*surA* and Δ*lpcA* (*p* < 0.05) (Figure 7), suggesting that the expression of adhesin and invasin in *E. coli* might be regulated by *surA* and *lpcA*.

## Discussion

We previously found that tosufloxacin (50 μM) had high activity against both *E. coli* and *S. aureus* persisters *in vitro* (Niu et al., 2015a,b), but it could not kill the biofilm persisters *in vivo* (unpublished observation). This means that persisters tolerant to tosufloxacin still exist, despite it is the most active quinolone against persisters in our previous study (Hongxia et al., 2018). In this study, using long exposure of 18 h with tosufloxacin (10 μM), we screened the *E. coli* KEIO mutant library to identify genes involved in persistence under tosufloxacin exposure. Though the KEIO collection had been used to identify persister related genes before, this is the first time it is used for identifying tosufloxacin tolerant persisters. We identified 18 mutants (*acrA, acrB, ddlB, dnaG, gltI, hlpA, lpcA, recG, recN, rfaH, ruvC, surA, tatC, tolQ, uvrD, xseA*, and *ydfI*) that failed to grow on LB plates after exposure with tosufloxacin (Table 2).

In contrast to using long exposure of 18 h with tosufloxacin as in this study, a previous study was done using short exposure of 6 h with ofloxacin to screen the same mutant library at stationary phase culture (Hansen et al., 2008). Within the identified mutants, apart from genes involved in DNA repair and recombination (*recG, xseA, xseB*) which were excluded from the previous study owing to increased sensitivity to ofloxacin, periplasmic molecular chaperone *surA* were identified in both screens. G*ltI, hlpA, ruvC, ddlB, ydfI*, and *tatC* have not previously been described as persister genes, and we found that their mutations caused defect in persistence exposure to tosufloxacin, indicating that these are unique genes involved in *E. coli* persistence to tosufloxacin. It is of interest to note that *recG, recN, lpcA, acr*A, and *acrB* were identified as genes related to rifampin induced antagonism or persister formation to ofloxacin by our group previously (Cui et al., 2018). The results also further confirm the phenomenon that different persister genes will be identified using different antibiotics at different antibiotic exposure times.

Furthermore, the deletion mutants in genes coding periplasmic proteins (*surA, lpcA, hlpA*, and *gltI*) had more defect in persistence to tosufloxacin than the other identified mutants, with *surA* and *lpcA* mutants being the most prominent. Although *surA* and *lpcA* were identified as possible persister genes before (Hansen et al., 2008; Cui et al., 2018), they have not been confirmed *in vivo* or *in vitro*. Thus, we chose *surA* and *lpcA* to further confirm their roles in *E. coli* persistence *in vivo* or *in vitro* separately. Our results showed that the persister phenotype showed more than 100–1,000-fold decrease in persistence against several stresses (antibiotics including tosufloxacin, ciprofloxacin, ofloxacin and gentamicin; acidic, hyperosmotic and heat conditions) for the *surA* and *lpcA* mutants than the wild type strain *E. coli* BW25113 *in vitro*. The “deep” persiser phenotype was further confirmed by complementation studies, and indeed the persistence phenotype of the complemented strains was restored to that of the wild type strain. Besides, both in the stationary phase bacteria and biofilm bacterial infection models, the *surA and lpcA* mutants had lower survival than the parent strain UTI89 *in vivo* in mice, and the stationary phase mutants had no or limited ability to infect kidney. Thus, our findings indicate that the *in vitro* identified persister mechanisms (*surA* and *lpcA*) are operative and valid for *in vivo* persisters, suggesting that proteins SurA, HlpA, GltI and LpcA are indeed important in the formation and survival of *E. coli* persisters not only *in vitro* but also *in vivo*. It is worth noting the unique genes identified in this study are mainly involved in periplasmic function of the cell, which may be related to the unique high activity of tosufloxacin against persisters. This is because the cell membrane is a known target of persister drugs (Hurdle et al., 2011). Future studies are needed to confirm this.

SurA was described as a parvulin-like peptidyl-prolyl isomerase (PPIase) and a periplasmic holdase chaperone, which facilitates the folding, assembling, delivery and maturation of β-barrel outer membrane proteins (OMPs) such as OmpA, OmpF, and LamB in Gram-negative bacteria (Thoma et al., 2015). In this study, we found that the persister phenotype of the *surA* mutant was decreased more than 100–1,000-fold in persistence to various antibiotics, acidic, hyperosmotic and heat conditions. The possible mechanism is that deletion of *surA* gene in *E. coli* may lead to a defect in outer membrane and thus decreased persistence of bacteria against antibiotics and other stresses *in vitro*. In addition, *surA* mutant had limited ability to form biofilm and decreased ability to attach and invade urothelial cells. Possible explanation for these phenomenons may be: (1) SurA functions as a chaperone for the outer membrane fimbrial usher FimD which is essential for type 1- and p-fimbriae (Justice et al., 2005). Accordingly, mutation in *surA* could influence the biogenesis of type 1-and p-fimbriae, which will impact the adherence and invasion of host cells. (2) Lack of *surA* in *E. coli* might induce the RcsCDB phosphorelay signaling pathway (Castanie-Cornet et al., 2006), which is required for biofilm development. Moreover, *surA* was still required for full virulence of *E. coli*, including the ability of the bacteria to compete with their host for iron (Vertommen et al., 2009), and insertion of LPS into the outer leaflet of the outer membrane (Bos et al., 2004). Thus, deletion of *surA* gene in *E. coli* UTI89, which decreased the pathogenicity and persistence of UTI89 bacteria *in vivo*, is consistent with its important role in maintaining fimbriae function. *HlpA* encodes periplasmic holdase chaperone Skp, which cooperates with *surA* in the biogenesis of β-barrel OMPs.In this study, we identified *hlpA* may be an important “deep” persister gene as *surA*.

Glutamate is important in the process of bacterial metabolism. *E. coli* contains several transporters for the uptake of glutamate, such as GltIJKL, GltP, GltS, and GadC. Among them, GltI is a known dual glutamate and aspartate periplasmic-binding protein which is a component of the sodium-dependent ABC transporter system (Willis and Furlong, 1975); GltP is a cytoplasmic membrane protein which is a carrier of an Na+-independent, binding protein-independent glutamate/aspartate transport system, but the GltP transport system is deficient in *E. coli* k12 strain (Deguchi et al., 1989); GltS is a carrier protein in cytoplasmic membrane which is a Na+-dependent, binding protein-independent glutamate transport system (Kahane et al., 1975); GadC is a glutamate antiporter in cytoplasmic membrane which regulates *E. coli* acid resistance (Richard and Foster, 2007). In this study, we found that *gltI* is involved in tosufloxacin-tolerant persister formation. In addition, we also found that *gltS* is involved in *E. coli* persister formation and survival both *in vitro* and *in vivo* (data not published). These findings reveal the pivotal role of glutamate metabolism in *E. coli* persistence. This finding is consistent with the previous observation in *S. aureus* where glutamate metabolism and transport have been found to be involved in persister survival (Yee et al., 2015, 2019a). The mechanism involved could be that glutamate metabolites such as NH3 allow bacteria to adapt to stress environments or work through regulating reactive oxygen species (ROS) generation by glutathione.

LpcA (also known as GmhA) is a soluble protein present in the cytoplasmic fraction, which functions as a phosphoheptose isomerase used in the biosynthesis of the core component of lipopolysaccharide (LPS) precursor glyceromannoheptose (Brooke and Valvano, 1996). The LPS of *lpcA* mutant lacks heptose (Tamaki et al., 1971) and the *lpcA* mutant had decreased persistence *in vitro* and *in vivo*, suggesting that the structural integrity of LPS is important for bacterial persistence and survival under stresses. We also found that *lpcA* mutant had limited ability to form biofilm and also had decreased ability to attach and invade urothelial cells (Figures 4, 7), suggesting the important role of bacterial LPS in biofilm formation and pathogenicity. Possible explanations for these phenomena may be: (1) mutant of *lpcA* may change the permeability barrier of outer membrane which prevents entry of small hydrophobic antibiotics (Leive, 1974; Nikaido, 2003), such as tosufloxacin, and allows Gram-negative bacteria to survive in stress environments, resulting in decreased persistence and survival under antibiotics or stresses. (2) LPS has the ability to induce expression of adhesion molecule and chemokines (Tanaka et al., 1997). Mutant of *lpcA* may change the structure of LPS, which will impact adherence and invasion and survival in the host cells.

In summary, we identified 18 mutants (*acrA, acrB, ddlB, dnaG, gltI, hlpA, lpcA, recG, recN, rfaH, ruvC,surA, tatC, tolQ, uvrD, xseA*, and *ydfI*) that failed to grow after exposure with tosufloxacin. Among these, G*ltI, hlpA, ruvC, ddlB, ydfI*, and *tatC* have not previously been described as persister genes. The *surA* and *lpcA* mutants are the most prominent in persistence defect as shown by *in vitro* and *in vivo* tests followed by *hlpA* and *gltI* mutants. The genes identified by this study provide new insight into the mechanisms of persister formation and maintenance under tosufloxacin. Our findings will likely provide novel therapeutic and vaccine targets for developing more effective treatment and prevention of persistent *E. coli* infections.

## Data Availability Statement

All datasets generated for this study are included in the article/supplementary material.

## Ethics Statement

The animal study was reviewed and approved by The Institutional Animal Care and Use Committee of Lanzhou University.

## Author Contributions

HN designed the study, performed part of the experiments, analyzed the data, and wrote the manuscript. TL mainly performed the experiments and analyzed the data. YZ conceived the work and revised the manuscript. JW, QC, FL, and JH performed part of the experiments. All authors agree to be accountable for the content of the work. All authors contributed to the article and approved the submitted version.

## Conflict of Interest

The authors declare that the research was conducted in the absence of any commercial or financial relationships that could be construed as a potential conflict of interest.
